# Hydroxyapatite Nanocoatings Deposited by Means of Resonant Matrix-Assisted Pulsed Laser Evaporation

**DOI:** 10.3390/ma17235778

**Published:** 2024-11-25

**Authors:** Dominik Maskowicz, Kacper Maroszek, Rafał Jendrzejewski, Mirosław Sawczak

**Affiliations:** Photophysics Department, The Szewalski Institute of Fluid-Flow Machinery, Polish Academy of Sciences, Fiszera 14, 80-231 Gdańsk, Poland; s180968pg@gmail.com (K.M.); rafj@imp.gda.pl (R.J.); mireks@imp.gda.pl (M.S.)

**Keywords:** hydroxyapatite, thin films, pulsed laser deposition, MAPLE, Ti6Al4V

## Abstract

Hydroxyapatite (HAp) is one of the most widely studied materials for utilization in the development of artificial implants. Research is mainly aimed at the production and modification of HAp coatings for simplification of the deposition process, cost reduction, and increase in biocompatibility. In this paper, the authors deposited HAp synthetic microparticles by means of matrix-assisted pulsed laser evaporation (MAPLE) on Ti6Al4V alloy plate substrates and obtained uniform HAp coatings without further treatment or modifications. The authors utilized a tunable pulsed laser to adjust its wavelength to the selected solvents, in order to optimize the process for deposition speed and quality. The following solvents were used as matrices: deionized water, isopropyl alcohol, and a 3:2 mixture of isopropanol:acetonitrile. Obtained coatings were examined by means of scanning electron microscopy, Raman spectroscopy, X-ray diffraction, and profilometry in order to evaluate coating quality, uniformity, and structural integrity. MAPLE deposition allowed the acquisition of approx. 200 nm thick coatings for water and isopropanol matrices and approx. 320 nm for isopropanol:acetonitrile matrix, which indicates an increase in deposition rate by 37%. The obtained coatings meet requirements for further biocompatibility testing, material modification, and composite synthesis.

## 1. Introduction

The development of metal-based orthopedic and dental implants leads to extensive research toward improving the mechanical and biological properties of these devices. Among the materials, titanium and its alloys became the focus of researchers. The increased use of titanium-based structures is related to its high mechanical and corrosion durability, good biocompatibility, and versatility. However, inorganic structures require additional treatment to adapt them as a hard tissue replacement. In the short term, reactions of the human body to foreign structures, including encapsulation by fibrous tissue, can significantly prolong the healing time. In the long term, contact with soft tissues can lead to corrosive and abrasive structural damage to the implant structure, metallic ions and fragments released to the organism, metallosis, and other side effects, including damage to adjacent joints and bones. One of the possible solutions to increase biocompatibility is to coat the implant surface with materials similar to bone tissues. Hydroxyapatite (CA_10_(PO_4_)_6_(OH)_2_, HAp) is considered one of the most attractive candidates. Research confirms that HAp significantly reduces the probability of fibrosis and increases adhesion between the implant and the host bone tissue [1,2,3].

So far, various methods have been investigated to deposit HAp coatings on metal surfaces. Among the most promising techniques are methods based on thermal spraying [4], sputter coating [5,6], dip coating [7], sol–gel coating [8,9], electrophoretic deposition [10], hydrothermal methods [11], and laser-based techniques such as pulsed laser deposition (PLD) [12,13] and matrix-assisted pulsed laser evaporation (MAPLE) [14]. Most of these methods require a high process temperature, which is required to obtain a crystalline structure of the layer. At the same time, high temperature can cause decomposition of the substrates used in the synthesis. Another problem in high-temperature synthesis is to match the thermal expansion coefficients of materials to avoid cracking of the coating.

Since each method requires choosing a trade-off between HAp crystalline phase uniformity, purity, coating thickness, and durability, many alternative methods of HAp coating fabrication have been extensively investigated. Pulsed laser deposition (PLD) is one of the most promising ones due to its good surface coverage, fast coating deposition, lack of chemical waste, and high process flexibility in terms of modifying the chemical composition and structure of the applied layer [15].

However, HAp coatings deposited by most methods (including PLD) are amorphous and require high-temperature (400–700 °C) processing to obtain a crystalline structure [16].

In this work, we propose the use of a variation of the laser deposition technique known as matrix-assisted pulsed laser evaporation (MAPLE) for the deposition of HAp coatings. Unlike the classic PLD technique, MAPLE is a low-temperature process. The material to be deposited in the form of a coating is presynthesized in a separate process and can then be applied to any surface in unchanged form. If the starting material has a nanocrystalline form, the deposited coating will also have such a structure without the need for additional thermal processing. This property also allows the application of crystalline coatings, e.g., on polymer or composite materials. In the MAPLE method, the material to be deposited as a coating is dissolved or suspended in an aqueous or organic solvent (matrix) with a concentration that usually does not exceed 5%. The solvent (matrix) is selected so that its laser radiation absorption coefficient is much higher than that of the material suspended in it. The solution or suspension is frozen at cryogenic temperature and then ablatively evaporated using a laser as in the classic PLD technique. In the ablation process, most of the laser radiation energy is absorbed by the matrix due to the higher absorption coefficient. The absorbed energy is transferred to the kinetic energy to the particles suspended in the matrix. The solvent itself evaporates, while the suspended materials are transferred to the substrate on which they form a thin layer. As mentioned above, to run the MAPLE process correctly, it is necessary to select the appropriate laser and matrix so that the absorption coefficient of the matrix radiation is much higher than that of the deposited material. For most solvents, the absorption coefficient is very small in the visible range of radiation, and its increase can be observed in the ultraviolet range, below 300 nm. In this range, the absorption coefficient also increases for most materials and therefore also for materials that we intend to deposit as a layer. There are known reports of the application of MAPLE for the deposition of biocompatible coatings for orthopedic structures based on HAp or its analogues or composites. In these publications, certain laser setups are used and are equipped with UV excimer lasers [17,18,19,20]. In the case of high-energy UV radiation, there is a risk of decomposition of some materials due to photochemical reactions. The situation changes in the near-infrared range, where vibrational bands of chemical bonds such as C-H and O-H appear, which are components of most organic solvents, as well as water. By selecting the laser wavelength in the near-infrared range, it can be tuned to a specific vibrational band, achieving resonant absorption.

To the authors’ knowledge, no report has been presented regarding the use of nanosecond infrared (IR) lasers in the MAPLE HAp coating process. The usage of a tuned IR laser in the MAPLE process has one significant advantage: most commonly used solvents (water [21], acetonitrile [22], dichloroethane, dichloromethane [23], and others) have poor absorption in the UV-Vis-NIR range of the most commonly used pulsed lasers (1064 nm and its harmonics: 532, 355, and 266 nm). In comparison, these solvents have an absorption coefficient of orders of magnitude higher in specific ranges of mid-infrared due to the presence of vibration resonance bands [24,25]. A proper tuning of the laser source significantly speeds up the deposition process and reduces the potential influence of the laser on the particles dispersed in the matrix. The authors previously presented an example of the use of a tuned nanosecond IR laser in the MAPLE technique for exceptionally fragile metalorganic structures [26]. This approach may not be essential for HAp itself, since there are no known reports of the destructive influence of pulsed laser on HAp nanoparticles; however, this method provides the possibility of including other fragile molecules towards the target, including biological structures such as proteins, which can prove to be an especially useful tool in examining and improving biocompatibility [27]. This opens the way for fast manufacturing of HAp biocomposites containing fragile components [28].

In this paper, a tuned mid-infrared (range: 2.5–3.5 μm) laser was used in the process of MAPLE in order to deposit coatings of HAp directly onto the Ti6Al4V alloy substrates. For the experiments, commercially available HAp nanoparticles were used and dispersed in selected solvents: deionized water (H_2_O), pure isopropanol (IPA), and the 3:2 mixture of isopropanol and acetonitrile (IPA + ACN). These dispersions were compared in terms of the sedimentation rate criteria of HAp particles and the quality of thin films deposited in the MAPLE process.

## 2. Materials and Methods

### 2.1. Substrates

**Figure 1:**
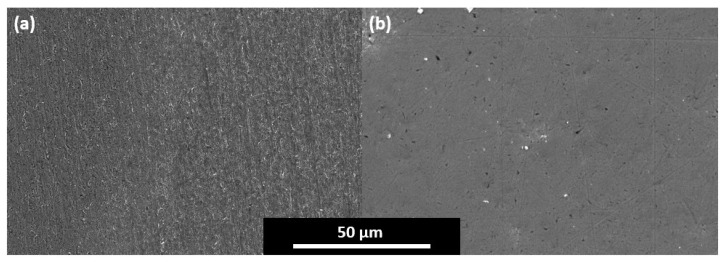
SEM images of the Ti6Al4V substrate (**a**) before and (**b**) after ceramic powder polishing.

The substrates for thin film deposition were prepared in the form of cuboid plates (10 × 15 × 2 mm^3^) made of Ti6Al4V alloy (grade 5, Bibus Metals, Dąbrowa, Poland) with percentage content of (89 ± 1)% Ti, (6 + 0.5)% Al, (4 ± 0.5)% V, and less than 0.5% of others (Fe, C, O, N, and H). The plates were cut down to the selected size from the larger rod, then ground on a metallographic plate grinder with 300-grit silicon carbide sandpaper. The next samples were polished using increasing grit: 500, 800, 1000, 1500, and 2000. Finally, the samples were polished by silica polishing powder (grit: 5000) and sonicated in acetone for 15 min to remove any metal and ceramic residue, then rinsed with isopropanol and dried with compressed air. The polishing results are presented in scanning electron microscope (SEM) images in Figure 1. In addition, silicon substrates prepared in the form of 10 × 5 × 0.3 mm^3^ plates were used for the deposition of reference samples for XRD and profilometry measurements. Si substrates were cleaned by sonication in acetone for 10 min, washed with isopropyl alcohol, and dried with air.

### 2.2. Hydroxyapatite Microparticles

**Figure 2:**
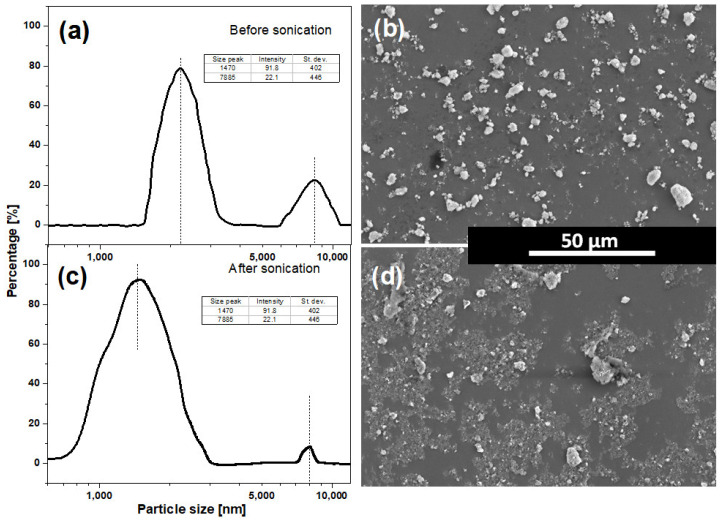
Size distributions (**a**,**c**) and SEM images (**b**,**d**) of HAp particles before (**a**,**b**) and after (**c**,**d**) sonication.

Commercially available HAp microparticles (Alfa Aesar, Thermo Fisher Scientific, Waltham, MA, USA) without any additional purification were used as a starting material. The powder was characterized using a Zetasizer Nano ZS with 632.9 nm laser source (Malvern Panalytical, Malvern, UK) and SEM imaging and the results are shown in Figure 2. As presented in Figure 2a, the measurements of the raw HAp nanoparticles indicate two major size fractions: larger (approximately 8.5 µm) and smaller (approx. 2.2 µm). Sonication of the suspension for 10 min significantly decreased the percentage of the largest particle fraction (23% → 8%), increased it for the smaller one (77% → 91%), and decreased the mean particle size in both fractions (8.5 µm → 8 µm and 2.3 µm → 1.5 µm, respectively)—Figure 2c. This effect was clearly confirmed by SEM imaging (Figure 2b,d). Further sonication did not cause any visible differences in particle sizes.

### 2.3. MAPLE Deposition

For MAPLE deposition, a custom experimental setup was used, which was described in the authors’ previous work [26]. The setup includes a vacuum chamber, turbomolecular pump with a pre-pump, a closed cycle helium cryostat (MicrostatN, Oxford Cryosystems, Long Hanborough, UK), a custom electromechanical setup to control substrate, and a target relative position and tuned mid-infrared laser equipped with a Nd:YAG pulsed laser as a main source and optical parametric oscillator (OPO) that can tune the incident laser beam in a wavelength range of 2.5–3.5 µm (Radiant X 2731 + 3034, Opotek, Carlsbad, CA, USA).

For the deposition process, the Ti6Al4V and silicon plates were attached to the substrate holder and placed inside the chamber. The chamber was pumped to the pressure of 10^−5^ mbar, then the cryostat was activated and the cryostat coolhead was cooled to 45 K. The vacuum pump was then temporarily switched off and the chamber was filled with dry nitrogen sourced from liquid nitrogen to the pressure of 500 mbar. After that, the target suspension was injected into the target container through a silicon tube and left to freeze. After target solidification, the vacuum pump was re-enabled back to 10^−5^ mbar.

The laser was tuned to 3080 nm for water and 2950 nm for isopropanol and the isopropanol:acetonitrile mixture, to match resonance absorption bands of the solvents. During deposition, the laser with a pulse energy of (9 ± 0.2) mJ was operated at a pulse repetition rate of 10 Hz. The intensity of the laser beam in the plane of the target surface was (0.8 ± 0.1) J/cm^2^. The target surface was scanned for 60 min (36 000 impulses) with constant rotation of the target container and substrate holder. After deposition, the vacuum chamber was filled with dry nitrogen and the samples were removed.

### 2.4. Sample Characterization

Raman analysis of deposited coatings was conducted by means of a micro-Raman spectrometer (InVia, Renishaw, Wotton-under-Edge, UK) equipped with a 514 nm laser source set up on a power of 0.2 mW focused by microscope optics on a circular spot having a 10 μm diameter. Raman spectra were recorded in a range of 200–1200 cm^−1^ with 5 accumulations. SEM analysis was performed with the SU3800 SEM microscope (Hitachi, Tokyo, Japan). The surface topography was analyzed using a DektakXT profilometer (Bruker, Billerica, MA, USA). XRF data were collected using a custom-built system equipped with an X-ray tube (Oxford Instruments, Long Hanborough, UK) operating at 55 kV (1 mA) and an SDD detector (Ketek, Munich, Germany) (155 eV resolution for Mn K_α_). XRD analysis was performed using an XRD (X’Pert PRO MPD, PANalytical, Almelo, The Netherlands) equipped with a Cu-Kα (1.541 Å) X-ray source.

### 2.5. Adhesion Test

Preliminary adhesion tests were conducted on Ti6Al4V coatings deposited on the IPA + ACN matrix. A x-cut tape test was used based on ASTM D3359-23 was used. The coating surface was cut using a freshly unpacked scalpel blade to form an ‘X’ cut on a surface to form an area of approximately 8 × 10 mm inside the cut. Next, a 15 mm wide piece of tape with an adhesive peel strength of 6.5 N/cm was placed on the surface to cover the sample surface. The tape surface was pressed with a piece of soft rubber to ensure uniform adhesion to the surface. After 90 s, the tape was removed and the samples were evaluated by comparison with the test classification.

### 2.6. Statistical Analysis

Statistical measurements of sample thickness, based on cross-sectional SEM and profilometry, were conducted on 5 separate sample batches for each matrix type. In the case of SEM, there was one sample (deposited on a Ti6Al4V substrate) for each batch and registered by SEM imaging. The sample thickness was estimated by measuring the sample thickness using Hitachi Image-Pro^®^ software version 2.3 (Hitachi, Tokyo, Japan) on 25 different spots (125 spots in total per sample type). In the case of profilometry, the samples deposited on silicon plates were used because of the much higher surface uniformity and flatness of the substrate. One sample for each batch was measured in 25 different spots (125 spots in total per sample type). The profile length was set to 2500 µm (approximately 500 µm of bare substrate surface and 2000 µm of sample surface. The thickness of the HAp coating for each type of sample was calculated by averaging the data obtained.

## 3. Results

### 3.1. Selection of Solvents

**Figure 3:**
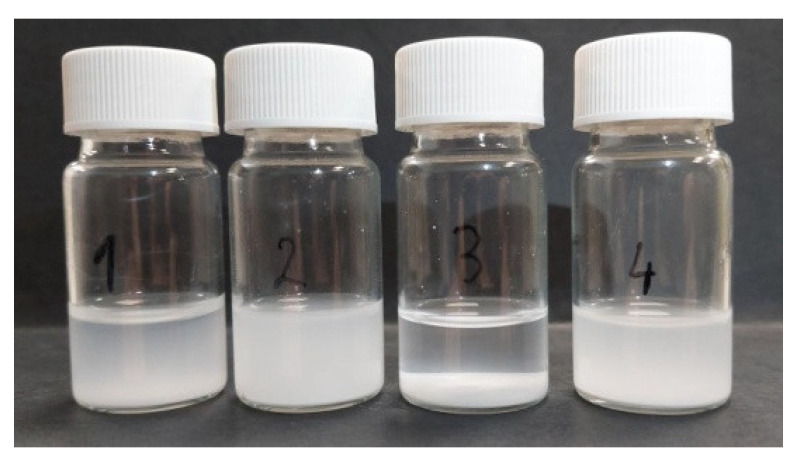
Sedimentation of HAp powder after 10 min in deionized water (1), isopropanol (2), acetonitrile (3) and 3:2 isopropanol:acetonitrile mixture (4).

HAp particles were dispersed in selected solvents, including deionized water, isopropanol (IPA), acetonitrile (ACN), and a 3:2 mixture of isopropanol and acetonitrile, to evaluate the sedimentation level. Each suspension was prepared by mixing 0.5 mL of liquid (approximately 0.5 g of water or 0.4 g of isopropanol, acetonitrile, or isopropanol:acetonitrile mixture) and HAp powder (20 mg in the case of water or 16 mg for other solvents) to obtain a mass concentration of 4%, followed by 10 min of sonication. The results after leaving for 10 min are presented in Figure 3.

In the suspension in deionized water (1), there is a slight sedimentation with a visible dispersed fraction. In this case, the process of sedimentation is sufficiently slow to counteract the quick solidification of water in cryogenic conditions because of the high melting point. The sample with a suspension of isopropanol (2) shows no visible sedimentation. However, the very low melting point of isopropanol (185.2 K), especially at the low pressure present in the MAPLE processing chamber, increased the difficulty of creating a stable target, which forced the authors to attempt utilizing acetonitrile (3), which has a higher melting point (228 K). In this case, the HAp particles created a sediment nearly instantly, which excluded it from further tests. To combine the advantages of both solvents, the authors decided to use a 3:2 mixture of isopropanol:acetonitrile (4), which turned out to sustain the suspension and has a reasonably high melting point of approx. 200 K.

### 3.2. MAPLE Deposition of HAp Coatings

#### 3.2.1. SEM Investigations

**Figure 4:**
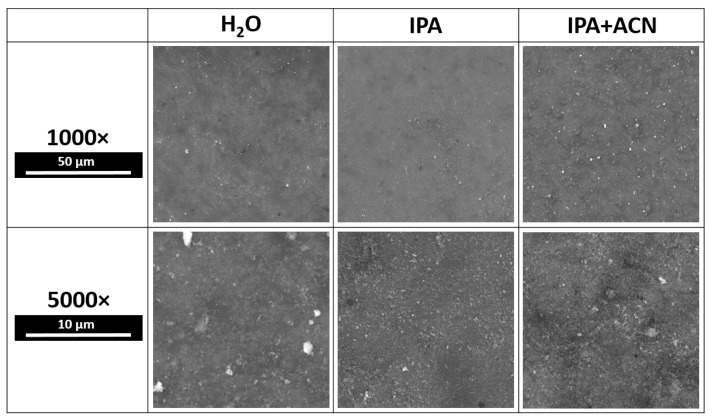
SEM images for HAp coatings synthesized by means of MAPLE with selected matrices.

Samples were deposited on Ti6Al4V alloy and Si substrates as described in the experimental section. SEM images of samples prepared using different solvents are presented in Figure 4.

In the first case of a water matrix, the sample is covered by irregular crystallites similar to the reference material. However, in the second and third cases, the structure appears to morph into a fibrous net-like unified structure, characteristic of unordered deposition without heat treatment. In each case, the samples are characterized with sufficient surface coverage. Although all images indicate a sufficiently uniform surface coating, IPA + ACN samples have the highest surface coverage and uniformity, while samples prepared with IPA reveal several patches of uncovered substrate with dimensions of several micrometers. Additionally, the IPA + ACN sample is more frequently covered by larger HAp aggregates, which are visible on SEM images as bright clusters. Brightness is caused by the charge accumulated from the SEM scanning beam, caused by poor contact with the formed coating. Due to their mass, these aggregates have the largest probability of being excluded from the suspension due to sedimentation, effectively decreasing the amount of deposited material and negatively influencing the coating thickness.

#### 3.2.2. Sample Thickness Investigations

**Figure 5:**
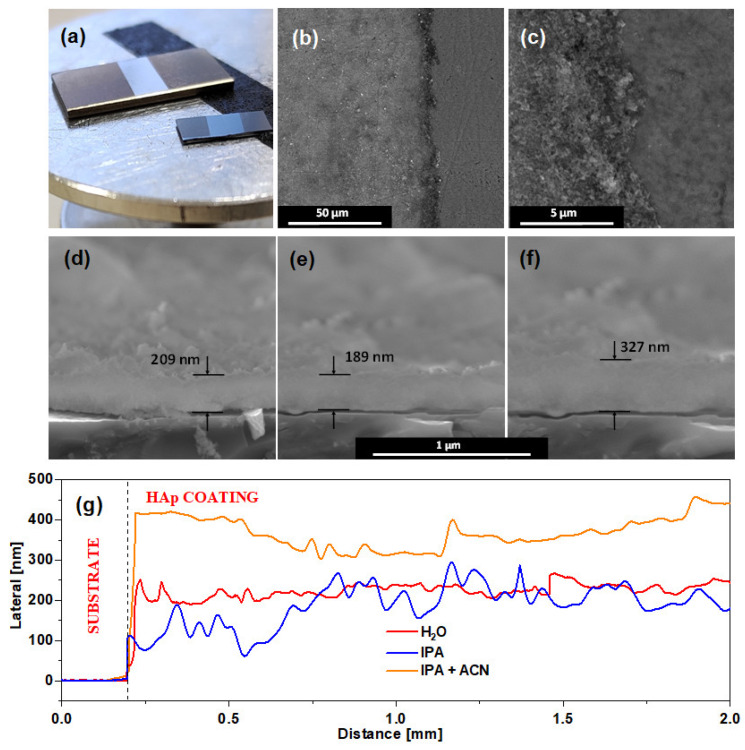
(**a**) Photograph of HAp coatings on Ti6Al4V substrate (larger) and silicon (smaller); (**b,c**) SEM images of exemplary coating surface borders deposited from IPA + ACN matrix on Ti6Al4V substrates; (**d**–**f**) cross-sectional SEM images with marked average layer thickness of HAp coatings on Ti6Al4V substrate deposited from (**d**) H_2_0, (**e**) IPA, (**f**) IPA + ACN matrices; (**g**) profilometry of synthesized HAp coatings.

The photographs of the HAp coatings deposited from the IPA + ACN matrix on the Ti6Al4V substrate and SEM images of the surface boundaries are shown in Figure 5a–c. The coating thicknesses were estimated by analyzing SEM cross-section images and profilometry measurements. The results of SEM analysis are presented in Figure 5 d–f. The average sample thickness was calculated as (209 ± 47) nm for H_2_O matrix samples, (189 ± 17.6) nm for IPA samples, and (327 ± 25.2) nm for IPA + ACN samples. The results of the profilometry are presented in Figure 5g. H_2_O matrix samples had average thickness x = (201.8 ± 16.9) nm with RMS = (86.1 ± 8.5) nm; IPA matrix samples had x = (169.1 ± 14.9) nm with RMS = (74.2 ± 6.3) nm; and IPA + ACN samples had x = (332.2 ± 27.3) nm with RMS = (115.5 ± 11.0) nm. The deposition rates were calculated as the number of pulses required to deposit a layer of 1 nm thickness. The results are presented in Table 1.

It is noticeable that the thickness in the samples is nearly one order of magnitude lower than the average size of the HAp particles obtained by means of Zetasizer measurements. This discrepancy is the result of the fact that the Zetasizer calculates the size of the particle by spheric estimation. As can be observed in SEM images of the particles from Figure 1, the irregular shape differs significantly from the spherical approximation. Additionally, nanosecond laser irradiation can cause aggregated particles; however, due to laser wavelength optimization of matrices and negligible absorption of HAp in the mid-infrared range [29], this effect has a minor impact on HAp particle size.

The results indicate that the IPA + ACN matrices guaranteed slightly lower relative roughness. Additionally, among these samples, the IPA + ACN matrix allowed the acquisition of HAp coatings that were almost twice as thick. The higher average roughness of the sample surface coincides directly with a presence of larger HAp particles. The evidence proves that among tested matrices in identical deposition parameters, the one containing 3:2 isopropanol:acetonitrile appears to provide the best HAp coating results on Ti6Al4V alloy substrate.

#### 3.2.3. Raman Investigations

**Figure 6:**
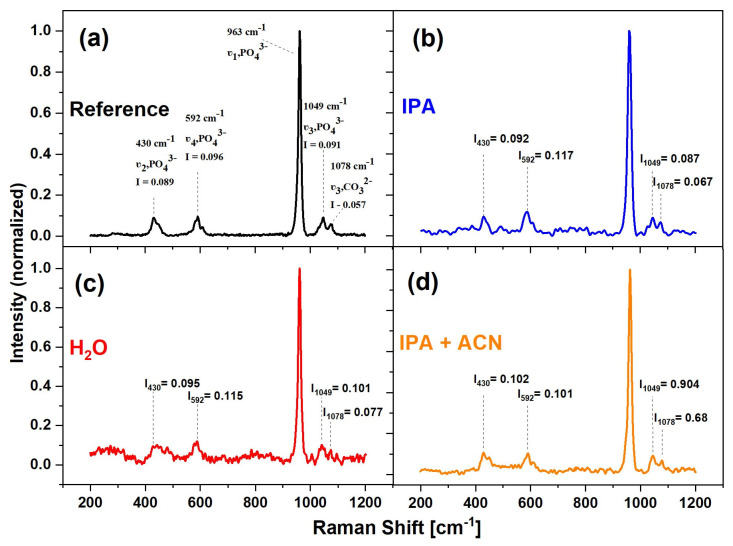
Comparison of Raman spectra of (**a**) reference HAp microparticles, synthesized HAp coatings using different matrices: (**b**) water, (**c**) isopropanol, and (**d**) isopropanol:acetonitrile mixture; normalized to 965 cm^−1^ band.

The samples were analyzed by Raman spectroscopy in order to evaluate the structure of HAp. The results are presented in Figure 6. In all three cases, Raman spectra reveal several bands important in the identification of HAp. These peaks will be used to identify HAp in MAPLE-deposited samples. The dominant peak of 963 cm^−1^ is correlated with the symmetric stretching mode of the P-O bond, the band around 430 cm^−1^ is related to the O-P-O ν_2_ bending mode, 592 cm^−1^ is related to ν_4_ the O-P-O bending mode, and 1078 cm^−1^ is associated with the ν_3_ antisymmetric stretching mode of the P-O bond. The wave numbers of the presented bands are in agreement with the data in the known literature [30]. The 1078 cm^−1^ band is interpreted as the presence of post-synthesis residual carbonate [31].

There are negligible discrepancies (and falling within the noise error) of relative intensities of the minor peaks, which means that coatings of all three samples retain the structure of crystalline HAp. However, differences in noise intensity in the samples, especially for samples prepared from an isopropanol:acetonitrile (IPA + ACN) matrix, indicate that the coating quality differs significantly. Any band-broadening effects related to alteration in crystallite structure (size, carbonate ion inclusion concentration, or presence of other inclusions) [32] can be observed; therefore, Raman spectra provide evidence that the MAPLE process did not cause modifications of the HAp structure in all examined cases.

#### 3.2.4. XRD Analysis

**Figure 7:**
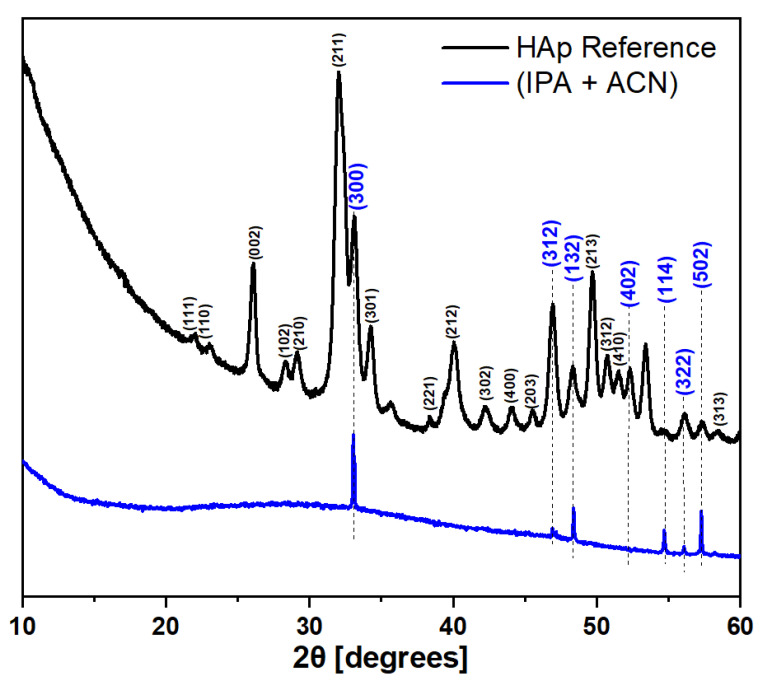
X-ray diffraction patterns of reference HAp microparticles (black) and MAPLE-synthesized coating on Ti6Al4V substrate (blue).

To analyze the crystallinity of the samples, X-ray diffraction was performed. The results are presented in Figure 7. As a reference sample, a drop-cast suspension of HAp particles on Ti substrate was used. Due to negligible differences between patterns of the MAPLE samples synthesized from different matrices, a single representative pattern from the IPA + ACN sample is presented. The XRD pattern of the reference sample represented as a black line agrees with other ones presented in other publications that present XRD of HAp nanoparticles [33,34,35]. On the other hand, the MAPLE-synthesized coating XRD pattern (blue line) significantly differs from the reference. Only a small part of the original pattern reflections is present. Among these reflections, there is a (300) reflection, which has the highest intensity in the reference pattern, and one (XX4) and five (XX2) reflections. The authors have encountered this effect in previous studies on the subject of MAPLE deposition [26]. HAp crystallites are more likely to attach to a substrate surface by the certain spatial configuration that creates a distinct texture effect [36,37]. Reinforcement of the reflections with particular Miller indices shows that crystallites are preferably placed on the substrate perpendicular to the c-axis of the hexagonal HAp unit cell.

#### 3.2.5. Adhesion Investigation

The X-cut adherence test of the tested samples was evaluated using ASTM D3359-23 classification. Among 10 tests, 6 resulted in 1A classification and 4 resulted in 0A classification. The result indicates poor sample adhesion compared to typical HAp coatings presented in the literature. As confirmed by Duta et al. [20], MAPLE-based HAp coatings require thermal treatment in order to obtain adherence values required by medical application. However, because the focus of the paper was deposition without thermal treatment, further studies are required in order to improve the adhesion of the presented samples.

## 4. Conclusions

In summary, a resonant infrared MAPLE technique was successfully applied for the fabrication of HAp coatings on Ti6Al4V. During 60 min of the deposition process, the Ti6Al4V alloy substrates were coated with a uniform layer of HAp originating from the suspension of synthetic microparticles. Among the three examined solvents utilized in the matrices for the deposition process, all three allowed the authors to obtain fair-quality coatings with the thickness of several hundreds of nanometers. However, analysis of the surface structure by means of scanning electron microscopy and profilometry indicates that a 3:2 mixture of isopropanol and acetonitrile provided the best coating quality among the options examined. Raman spectroscopy and X-ray diffraction analysis indicate that the deposition process did not have a negative impact on the crystalline structure of the HAp particles. Although the mechanical properties of the investigated coatings appear to be inferior to those synthesized by means of other common methods reported in the literature, the technique provides a possibility of non-destructive co-deposition of fragile biocomponents that can additionally functionalize HAp coatings. This advantage will be considered in further research towards the application of MAPLE in HAp nanolayer production.

## Figures and Tables

**Table 1:** Deposition rates calculated from the average sample thickness estimated by cross-sectional SEM images and profilometry.

Sample (Matrix)	Deposition Rate (SEM) [Impulse/nm]	Deposition Rate (Profilometry) [Impulse/nm]
H_2_O	190.5 ± 18	179 ± 16
IPA	172 ± 16	213 ± 19
IPA + ACN	112 ± 10	108 ± 9

## Data Availability

The data presented in this study are available from the corresponding author on request.
